# Significant Association of *DNASE1* Variable Number Tandem Repeats and Single Nucleotide Polymorphisms With Gastric Cancer

**DOI:** 10.3389/bjbs.2022.10526

**Published:** 2022-05-13

**Authors:** Ali Kafil, Parisa Mohamadynejad, Mehdi Moghanibashi

**Affiliations:** ^1^ Department of Biology, Faculty of Basic Sciences, Shahrekord Branch, Islamic Azad University, Shahrekord, Iran; ^2^ Department of Genetics, School of Medicine, Kazerun Branch, Islamic Azad University, Kazerun, Iran

**Keywords:** gastric cancer, *DNASE1* gene, VNTR, polymorphism, intron, exon

## Abstract

**Introduction:** Defects in the apoptotic process are among the most important events involved in carcinogenesis, and defects in *DNASE1*, as one of the apoptotic machinery components, plays a role in various types of cancer. Previous studies have indicated significant differences in the *DNASE1* polymorphisms in different populations. We hypothesized an association of two polymorphic sites in the exon 8 and the intron 4 of the *DNASE1* gene with the risk of gastric cancer.

**Materials and Methods:** The study was carried out on 120 gastric cancer patients and 120 age and sex adjusted controls using PCR and RFLP-PCR.

**Results:** The genotype GG (rs1053874) in exon 8 of *DNASE1* (odds ratio [95% confidence interval]) 4.65 [2.10–10.29], *p* < 0.001) and genotype 2/3 of variable number tandem repeat (VNTR) in the intron 4 (3.75 [1.56–9.01], *p* = 0.003) are both linked to gastric cancer.

**Conclusion:** We propose that both polymorphic sites have a part to play in gastric cancer, and so may be useful diagnosis markers.

## Introduction

Gastric cancer is the fourth most common cancer and the second leading global cause of cancer mortality (1), whose incidence varies greatly in different parts of the world (2). Despite the recent decrease in the incidence of gastric cancer in many parts of the world, its incidence is increasing in other parts of the world, including Iran, where gastric cancer is responsible for 14% of all causes of cancer death (3). Risk factors such as *helicobacter pylori*, high levels of salt-preserved foods intake, tobacco smoking, and pernicious anemia, do not fully explain susceptibility (4–10), suggesting other as yet undiscovered factors, such as genetics. *DNASE1*, the gene which encodes the enzyme DNase I, is located on chromosome 16, locus p13.3 and consists of nine exons and eight introns (11–13). It is expressed in different organs including the stomach (14–17). Numerous studies have provided evidence on the role of DNase I in the endonucleolytic process of apoptosis and its role in various cancers (18, 19, 12, 14). In the previous studies, significant variation in *DNASE1* polymorphisms have been identified in different populations (20, 21), one being the *DNASE1* 1*2 polymorphism (rs1053874) that is also conserved in other mammals (20). It is an A nucleotide transition to G nucleotide occurring at position 2317 in the exon 8 and results in a Gln to Arg substitution at amino acid position 222 of the mature enzyme (Gln222Arg) (22–25). A 56-bp variable number of tandem repeats (VNTR) with five alleles designated as HumDN1 is another polymorphism in the *DNASE1* located in the intron 4 (11, 20, 26, 27).

Considering the role of *DNase I* enzyme in the apoptosis and the effects of genetic polymorphism on its activity (15), we hypothesized links between the A2317G polymorphism and the 56-bp VNTR (HumDN1) in *DNASE1*, and gastric cancer.

## Materials and Methods

A whole blood sample was obtained from 120 patients with gastric cancer who were referred to Omid Hospital (Isfahan, Iran) during the period of 2013–2015. Diagnosis of the gastric cancer was confirmed histologically by tissue examination at the pathology department and tumors were classified according to Lauren’s classification (28, 29). Patients were aged (mean ± SD) 58.9 ± 12.1 and included 37 women and 83 men; informed consent was obtained from all participants. Controls were 120 healthy subjects aged 58.2 ± 12.9 and gender matched (*p* = 0.69), who were randomly selected from volunteer blood donors. The latter were in complete heath, without any history or signs of gastric cancer or previous gastric medical complications, and all subjects were unrelated. The ethics and study protocol were approved by both the Omid Hospital ethics committee and the research ethics board of the Shahrekord Islamic Azad University.

**FIGURE 1:**
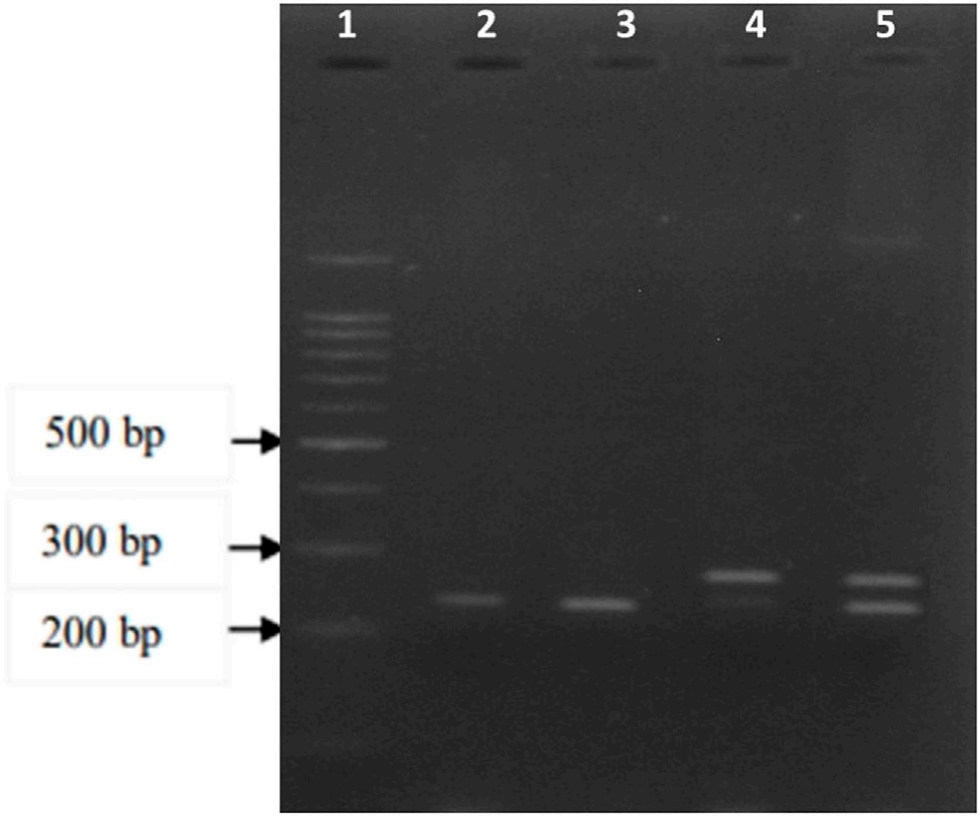
Electrophoresis of the A2317G polymorphism PCR amplified products on a 3.5 percent agarose gel following the digestion with XhoI restriction enzyme. Well 1 represents the 100 bp DNA ladder, well 2 and 3 are genotype GG (234 bp), well 4 is genotype AA (260 bp), and well 5 is the genotype AG (234 and 260 bp).

**FIGURE 2:**
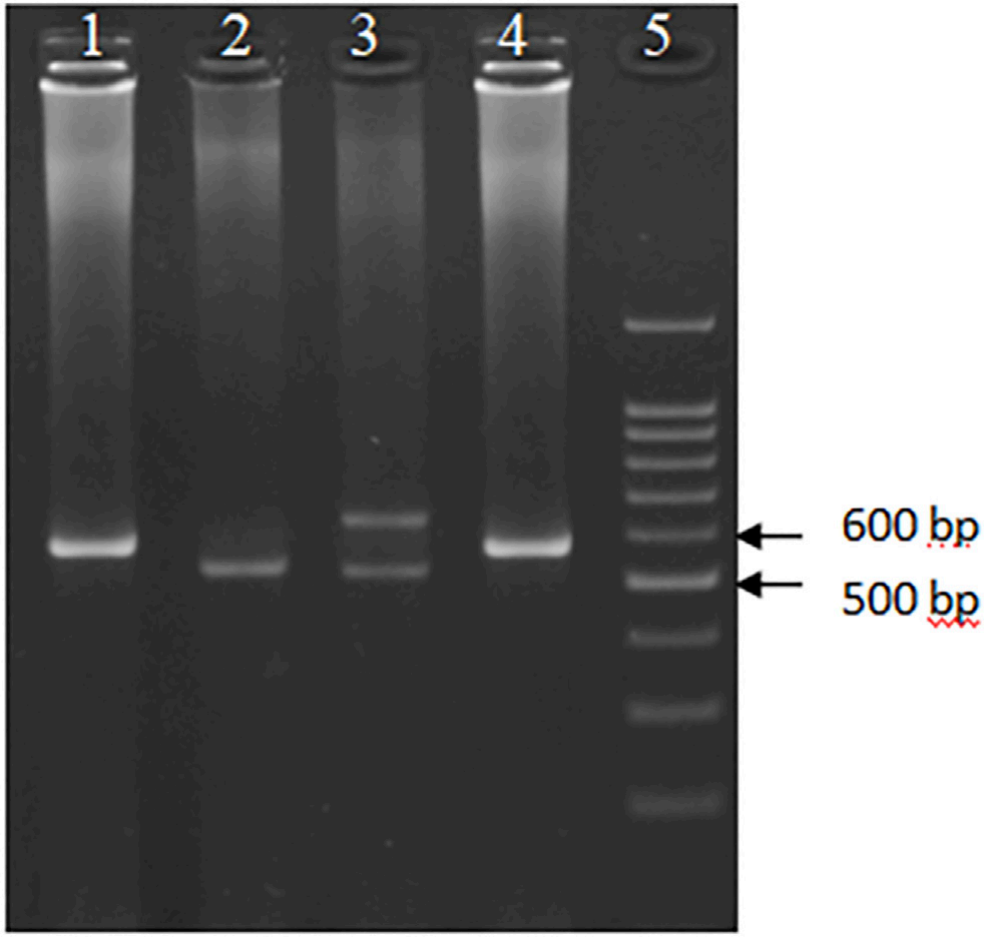
Electrophoretic pattern of the PCR amplified HumDN1 polymorphism on a 3.5 percent agarose gel. Wells 1 and 4 are genotype 3/3 (573 bp), well 2 genotype 2/2 (517 bp), well 3 genotype 4/2 (517 and 629 bp), and well 5 represents size marker (DNA ladder 100 bp).

Genomic DNA was extracted from the whole blood samples using salting out method (30). The genotype of the *DNASE1* 1*2 polymorphism was assessed using Polymerase Chain Reaction- Restriction Fragment Length of Polymorphism (PCR-RFLP). Firstly, the genomic region including the target SNP was amplified using primer pair; 5′- ATC​GTG​GTT​GCA​GGG​ATG​CTG​CCT​C-3′ (forward) and 5′-AGT​TCA​ACA​GGT​GTG​GGG​AG-3′ (reverse). PCR was performed in a 20 µl reaction mixture containing 5 ng of the target DNA, 1X buffer (15 mM Tris–HCl, pH 8.0, 50 mM KCl), 1.5 mM MgCl_2_, 5 pM of each primer, 200 µM dNTPs, and 1 U of Taq polymerase. All amplifications were performed following the 5 min initial denaturation at 94°C and 35 cycles of subsequent amplification according to the following scheme: 1 min at 94°C, 30 s at 54.2°C, 30 s at 72°C, followed by a 7 min final extension at 72°C. Secondly, the PCR products were digested with the restriction enzyme XhoI (Fermentas) at 37°C for 3 h based on the outlined protocol of the kit. The presence of XhoI site was identified by the cleavage of the 260 bp amplified product to yield fragments of 234 and 26 bp on a 3.5% agarose gel (Figure 1). The other polymorphism, HumDN1, was assayed using PCR technique by primer pair 5′- GCA​CCA​GAC​ACC​TAT​CAC-3′ (forward) and 5′-CAT​CGT​AGT​AGT​AGC​TGT​CC-3′ (reverse), which was amplified 573 bp for the allele with three repeats (Figure 2). PCR was performed in the same condition except with 28 cycles and 60°C for the annealing step. Finally, electrophoresis was performed on a 2% agarose gel. In this study, ethidium bromide was replaced with the DNA Stain Safe View I (KIAGENE, Iran) for gel visualization. Ten percent of the samples were randomly selected and the assay was repeated; results indicated 100% concordance.

Hardy–Weinberg equilibrium was assessed from the values obtained by χ2 test. The association between the polymorphisms and gastric cancer was evaluated by odds ratios (OR) and their 95% of confidence intervals (CI), as calculated using unconditional logistic regression models. *p* < 0.05 was considered significant and all tests were two-sided. Analysis was performed using IBM SPSS 17 software.

## Results

The frequencies of the alleles and the genotypes are presented in Table 1. All the genotypic distributions were consistent and in accordance with the Hardy–Weinberg model. For HumDN1 polymorphism, all the previously reported alleles, except alleles with five repeats, were considered in our study. Most HumDN1 alleles were found to be alleles with the two repeats (37.9%) and three repeats (40%) in the controls and cases, respectively. The majority of HumDN1 genotypes in the controls and in the cases were 3/4 (20%) and 2/3 (35.8%), respectively. Overall, the genotype 2/3 of HumDN1 was associated with gastric cancer risk (*p* = 0.003). Although genotype 3/3 was associated with a higher likelihood of intestinal type of gastric cancer (*p* = 0.04), there is a wider confidence interval because of the small sample size in each group. Generally, an allele with three repeats was significantly associated with gastric cancer (*p* = 0.008). For the *DNASE1* 1*2 polymorphism, subjects with the genotype GG were significantly linked with gastric cancer (*p* < 0.001). Generally, we found that allele G is significantly associated with gastric cancer (*p* < 0.001). Interestingly, a combination of the HumDN1 and *DNASE1* 1*2 polymorphisms analysis (Table 2) revealed an increased link of gastric cancer with GG-2/3 genotype (*p* = 0.008). However, there is a wider confidence intervals because of the small sample size in each group after combination of two polymorphisms.

**TABLE 1:** Genotype and allelic frequencies of the *DNASE1* polymorphism in gastric cancer patients and the healthy controls.

SNP	Variant	Cases (%)	Controls (%)	OR	95% CI	*p value*
Rs1053874	AA	51 (42.5)	72 (60)	Ref	—	—
	AG	36 (30)	38 (31.7)	1.33	0.74–2.38	0.32
	GG	33 (27.5)	10 (8.3)	**4.65**	2.10–10.29	**<0.001**
	AG + GG	69 (57.5)	48 (40)	**2.02**	1.21–3.39	**0.007**
	A	138 (57.5)	182 (75.8)	Ref	—	—
	G	102 (42.5)	58 (24.2)	2.32	1.92–2.81	**<0.001**
VNTR	2-2	12 (10)	22 (18.3)	Ref	—	—
	3-3	16 (13.3)	12 (10)	2.44	0.87–6.82	0.08
	4-4	11 (9.20)	15 (12.5)	1.34	0.47–3.83	0.58
	2-3	43 (35.8)	21 (17.5)	**3.75**	1.56–9.01	**0.003**
	3-4	21 (17.5)	24 (20)	1.60	0.64–4.01	0.31
	2-4	17 (14.2)	26 (21.7)	1.19	0.47–3.04	0.70
	2	84 (35)	91 (37.9)	Ref	—	—
	3	96 (40)	69 (28.7)	**1.51**	1.22–1.86	**<001**
	4	60 (25)	80 (33.3)	0.81	0.65–1.01	0.06

Bold indicates significant data (lower than 0.05 in p value column and the increase risk corresponding to it in OR column).

**TABLE 2:** The combined genotype frequencies of the SNP and VNTR assessment in *DNASE1*; both in the gastric cancer patients and the healthy controls.

Genotype	Cases (%)	Controls (%)	OR	95% CI	*p value*
AA/2-2	7 (5.8)	12 (10)	Ref	—	—
AA/3-3	9 (7.5)	6 (5)	2.57	0.64–10.33	0.18
AA/4-4	3 (2.5)	11 (9.2)	0.46	0.09–2.27	0.34
AA/2-3	20 (16.7)	10 (8.3)	**3.42**	1.03–11.41	**0.04**
AA/3-4	5 (4.2)	16 (13.3)	0.53	0.13–2.10	0.37
AA/2-4	7 (5.8)	17 (14.2)	0.71	0.19–2.54	0.59
AG/2-2	2 (1.70)	8 (6.70)	0.42	0.07–2.61	0.35
AG/3-3	5 (4.20)	5 (4.20)	1.71	0.36–8.08	0.49
AG/4-4	5 (4.20)	3 (2.50)	2.85	0.51–15.76	0.22
AG/2-3	11 (9.2)	10 (8.30)	1.88	0.43–6.68	0.32
AG/3-4	9 (7.5)	5 (4.20)	3.08	0.73–12.98	0.12
AG/2-4	4 (3.3)	7 (5.80)	0.98	0.21–4.57	0.97
AG/2-2	3 (2.5)	2 (1.7)	2.57	0.34–19.33	0.35
AG/3-3	2 (1.7)	1 (0.80)	3.42	0.26–45.02	0.34
AG/4-4	3 (2.5)	1 (0.80)	5.14	0.44–59.45	0.19
AG/2-3	12 (10.0)	1 (0.80)	**20.57**	2.18–193.79	**0.008**
AG/3-4	7 (5.8)	3 (2.5)	4.00	0.77–20.67	0.09
AG/2-4	6 (5.00)	2 (1.7)	5.14	0.80–32.77	0.08

Bold indicates significant data (lower than 0.05 in p value column and the increase risk corresponding to it in OR column).

## Discussion

Since only a fraction of people exposed to the risk factors develop gastric cancer, genetic susceptibility (polymorphisms for example) may play an important role. Recently, Mocellin et al performed a systematic review and meta-analysis and found that certain variants in different genes were associated with susceptibility to gastric cancer (31). We hypothesized that both polymorphisms in *DNASE1* are linked with gastric cancer, by virtue of roles in apoptosis (17).

We found that genotype GG is significantly associated with an increased risk of gastric cancer (i.e., allele G is significantly associated with the gastric cancer development), which it is in line with previous studies (12, 14). Although the specific activity of phenotype 2 of *DNase I* enzyme is very similar to that of phenotype 1 (14, 15), there is evidence indicating a difference in the secondary structure of two forms of enzyme due to the difference between amino acids Gln and Arg at this polymorphic site (14, 15). In addition, phenotype 2 is more unstable than phenotype 1 under harsh conditions (14, 15). Therefore, it is reasonable to propose that this polymorphism could be a cause of predisposition and susceptibility to gastric carcinoma formation.

Regarding HumDN1 polymorphism, the genotype 2/3 (heterozygote genotype with the two and three repeats alleles) was associated with a higher likelihood of gastric cancer. According to a previous study that genotype 2/3 of the HumDN1 (56-bp VNTR) polymorphisms may affect the activity of enzyme (15), it is reasonable to propose that this polymorphism could be a cause of predisposition and susceptibility to the gastric cancer. Therefore, both polymorphisms may affect *DNase I* activity, which in turn, might cause a decrease in apoptosis and an increased risk of progression of gastric cells to acquire cancerous phenotype. Thus, we speculate a role for the functional alterations in the enzyme by means of HumDN1 and *DNase1* 1*2 (rs1053874) polymorphisms in gastric cancer susceptibility.

This paper is an advance in biomedical science as it points to links between polymorphisms in *DNASE1* and gastric cancer, which may be useful in diagnosis.

## Data Availability

The original contributions presented in the study are included in the article/supplementary material, further inquiries can be directed to the corresponding author.
